# Automated Classification of Benign and Malignant Proliferative Breast Lesions

**DOI:** 10.1038/s41598-017-10324-y

**Published:** 2017-08-29

**Authors:** Evani Radiya-Dixit, David Zhu, Andrew H. Beck

**Affiliations:** 1 The Harker School, Department of Pathology, Beth Israel Deaconess Medical Center and Harvard Medical School, Boston, CA 95128 MA USA; 2 Department of Pathology, Beth Israel Deaconess Medical Center and Harvard Medical School, Boston, MA USA

## Abstract

Misclassification of breast lesions can result in either cancer progression or unnecessary chemotherapy. Automated classification tools are seen as promising second opinion providers in reducing such errors. We have developed predictive algorithms that automate the categorization of breast lesions as either benign usual ductal hyperplasia (UDH) or malignant ductal carcinoma *in situ* (DCIS). From diagnosed breast biopsy images from two hospitals, we obtained 392 biomarkers using Dong *et al*.’s (2014) computational tools for nuclei identification and feature extraction. We implemented six machine learning models and enhanced them by reducing prediction variance, extracting active features, and combining multiple algorithms. We used the area under the curve (AUC) of the receiver operating characteristic (ROC) curve for performance evaluation. Our top-performing model, a Combined model with Active Feature Extraction (CAFE) consisting of two logistic regression algorithms, obtained an AUC of 0.918 when trained on data from one hospital and tested on samples of the other, a statistically significant improvement over Dong *et al*.’s AUC of 0.858. Pathologists can substantially improve their diagnoses by using it as an unbiased validator. In the future, our work can also serve as a valuable methodology for differentiating between low-grade and high-grade DCIS.

## Introduction

Pathologists must identify precursor lesions as either benign usual ductal hyperplasia (UDH) or malignant ductal carcinoma *in situ* (DCIS) for diagnosis and treatment of breast biopsies. Most patients with UDH receive no treatment and have minimal or no increased risk of cancer, while patients with DCIS are more likely to be diagnosed with invasive breast cancer^1, 2^. Treatment to reduce DCIS recurrence and invasive carcinoma has notable risks and side effects, given the extensive methods of lumpectomy with radiation, mastectomy, and tamoxifen hormonal treatment^3^. Diagnostic oversights can lead to either untreated cancer or unnecessary radiation treatment and chemotherapy, both of which have detrimental consequences. Thus, accurate diagnosis is critical for patients as well as for hospitals to reduce extraneous treatment costs. However, human pathologists may not always be in concordance as there is no strict set of instructions on how to carry out a diagnosis. In a study by Jain R.K. *et al*.^4^, researchers found that nine pathologists were in complete agreement in only 9 of 81 total cases of UDH and DCIS. Therefore, given the extreme treatment disparity between these two classes and the limited number of trained pathologists available, a second opinion based on an automated model would help reduce bias and variability and improve tumor diagnosis reliability by identifying challenging diagnosis cases.

Currently, computational pathologists identify morphological features from precursor lesions and apply statistical models on those features for lesion type discrimination. The lesion features are extracted through whole-slide image digitalization with multiplexed antibody stains^5^, image segmentation, and measurements of features such as nuclear area and perimeter^6^. These methods are primarily used in existing bioinformatics cancer research, and pathologists in the clinical setting typically use single-marker immunohistochemistry^7, 8^.

Dong *et al*. computationally extracted features from diagnosed tissue images, which were then used as input to an L1-regularized logistic regression machine learning model^6^. This model was successfully trained to differentiate between UDH and DCIS, and as a consequence of using the L1 regularizer, active features were obtained from the given input.

**Figure 1:**
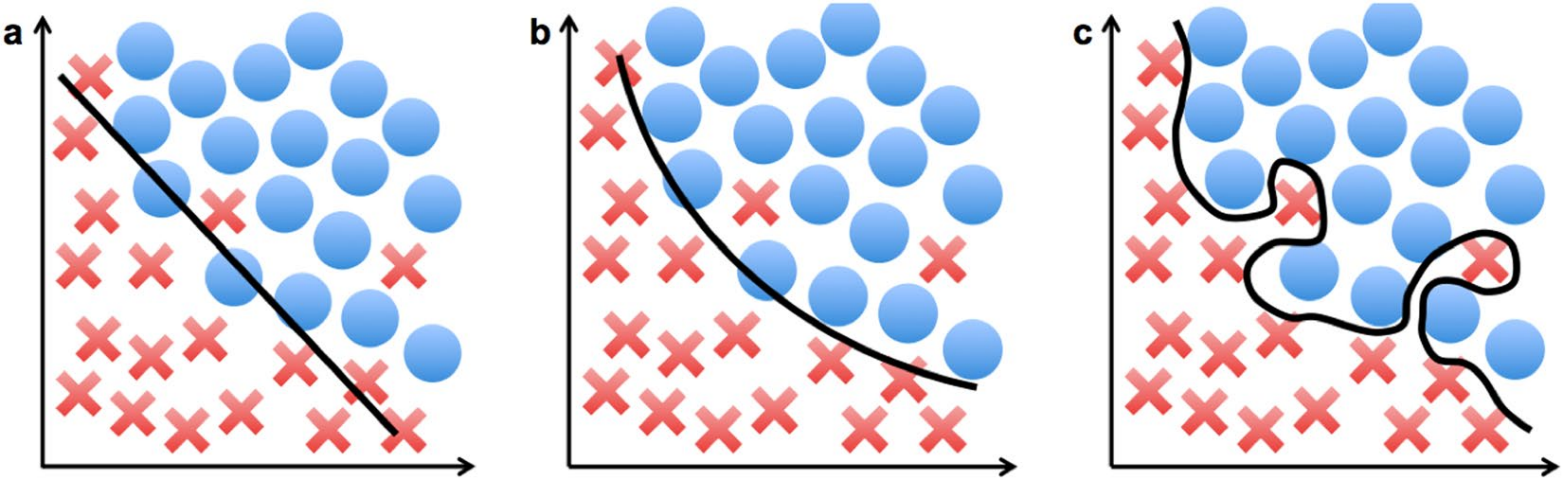
(**a**) An underfitting model. (**b**) An ideal model that identifies the underlying relationship of the data. (**c**) An overfitting model.

Despite advancements in techniques described by existing literature, there are still various limitations and areas for improvement in feature extraction and model selection. First, a manual intervention is necessary for feature extraction, especially for segmentation and nuclear tracing^6^. Second, existing models often have redundant training data of the morphological features, so active features should be identified and utilized to refine the algorithm input. Third, previous studies tended to focus on data collection and feature extraction and have not compared and combined multiple algorithms for better prediction. Leveraging prediction results from several models would be potentially useful for accuracy improvement. Finally, many existing models have not been applied to validation datasets^9^. They may not generalize well to datasets obtained from different hospitals, causing the algorithms to overfit and describe the noise of the training data instead of identifying the underlying relationship as shown in Fig. 1.

To strengthen the discrimination between DCIS and UDH, we enhanced prior work in a number of ways. We implemented six different machine learning methods to differentiate between the two diagnostic categories using automatically extracted cellular features. The diverse set of computations enabled a clearer understanding of the correlation between the quantitative features and breast lesions. We then curated the features dataset to keep only the active features by eliminating features that were not pertinent to the classification. We also combined algorithms, a strategy that has not been used previously in lesion categorization to our knowledge^6, 9–12^. Using diagnosis predictions from multiple models, we reduced prediction randomness and greatly improved accuracy. Finally, we validated the methods by training and testing them with datasets from different hospitals. Achieving high accuracy when we train our model with data from one hospital and test it with data from another indicates that our model makes predictions independent of hospital-specific data curation and would be able to generalize well to datasets from other hospitals. By reducing interobserver variability^4^, our automated investigational tools show potential in aiding pathologists with breast cancer decision support, serving as valuable, unbiased validators. The model can be used in different clinical studies across institutions.

## Results

First, to compare our study to previous work, we reran Dong *et al*.’s algorithm with 1000 seeds to account for prediction randomness. Their model had been evaluated on the same MGH and BIDMC datasets and achieved a validation score of 0.858. Next, we ran our six machine learning models on all 392 features, evaluating each with the AUC-ROC values. Most validation scores improved when the models were run on the refined active features dataset. By further combining the two logistic regression algorithms, we obtained our top-performing model with a validation score of 0.918.

### Scoring of Statistical Models

We obtained two scores to represent each algorithm’s performance. A validation score (V-score) was obtained by training the model on the 116 samples from the MGH hospital and testing its accuracy on the 51 samples from the BIDMC hospital. A cross-validation score (C-score) was obtained from training and testing with ten folds on all 167 samples from two hospitals. Since it is more applicable than the C-score, we used the V-score for comparison of our model performances and those from other studies. Achieving a high V-score is difficult as it represents the algorithm’s ability to extrapolate to datasets from other hospitals.

For each model, we evaluated its score by computing the AUC of the ROC curve, which was created from the model’s predictions on the test dataset^13^. An ROC curve provides a more accurate scoring measure than a simple true/false ratio since it accounts for the degree of confidence of a prediction between 0 and 1.

### Recalculation of Results from Existing Work to Account for Randomness

Dong *et al*. had developed a statistical model using the L1-regularized logistic regression model on the same MDH and BIDMC cases. The study had used only a single seed for computing C-scores and V-scores and thus did not reduce the randomness that resulted from the automated fold selection. We reran their models using 1000 seeds to reach more accurate AUC values for analysis and comparison to our own data. Dong *et al*.’s algorithm achieved a C-score AUC of 0.931, which was equivalent to our initial C-score without active features. The model achieved a V-score AUC of 0.858^6^.

### DCIS/UDH Classification Models

For six machine learning models, we obtained the C-score for classification with all features and with active features as well as the V-score for classification with active features. Table 1, column 2 includes the C-score AUC-ROC values of these algorithms for discrimination between DCIS (100 cases) and UDH (67 cases).

**Table 1:** The performances of the six machine learning models with all features (column 2) and with the active features (column 3) in terms of the AUC.

Algorithm	C-score for classification with all features	C-score for classification with active features	V-score for classification with active features
L1-regularized LR	0.931	0.921	0.897
LR w/early stopping	0.904	0.923	0.884
Random forest	0.854	0.878	0.666
Convolutional neural network	0.779	0.850	0.650
Conditional inference forest	0.801	0.822	Did not run
Multi-layer perceptron	0.695	0.489	Did not run

The V-scores for classification with the active features (column 4) indicate each model’s generalizability. We used 1000 seeds to account for the random number variance.

### Analyses of DCIS/UDH Classification Models with Active Features

We also obtained the C-score for the six machine learning models for classification with active features (Table 1, column 3). For each fold, the training data was used for the selection of the active features, and the features dataset was revised accordingly. Almost all algorithms had an increase in AUC, revealing the accuracy of the selected active features and the improved performance when irrelevant features were eliminated.

We evaluated the four top-performing algorithms by training the predictive models with active features on the MGH samples and testing those fixed models on the BIDMC samples (Table 1, column 4). The active features from training the L1-regularized logistic regression model on the MGH cases were used again for the V-scores of all algorithms. We obtained high performances for the L1-regularized logistic regression and logistic regression with early stopping models (AUC = 0.897 and 0.884, respectively, about 3% higher than the AUC V-score of 0.858 achieved by Dong *et al*.). However, the V-scores for the random forest and convolutional neural network algorithms were less impressive (AUC = 0.666 and 0.650, respectively). These results indicate that logistic regression models displayed stronger generalizability than decision tree-based learning methods when running each algorithm on different, unseen data from an independent source.

### Analyses of the Combination of Top-performing DCIS/UDH Classification Models

The logistic regression with early stopping and L1-regularized logistic regression had high C-scores and V-scores for classification with active features. To verify our hypothesis that strong prediction models tend to correct each other, we combined them to create our CAFE (Combined with Active Feature Extraction) model. The scores for the individual logistic regression algorithms and the CAFE model are listed in Table 2. For comparison, we added the scores of Dong *et al*.’s model^6^.

**Table 2:** The performance of Dong *et al*.’s model, our two LR algorithms with active features, and our CAFE model.

Algorithm	C-score	V-score
L1-regularized LR from Dong *et al*.	0.931	0.858
L1-regularized LR with active features	0.921 (SD of 0.0064)	0.897
LR with early stopping and active features	0.923 (SD of 0.0020)	0.884
CAFE model	0.921	0.918

**Figure 2:**
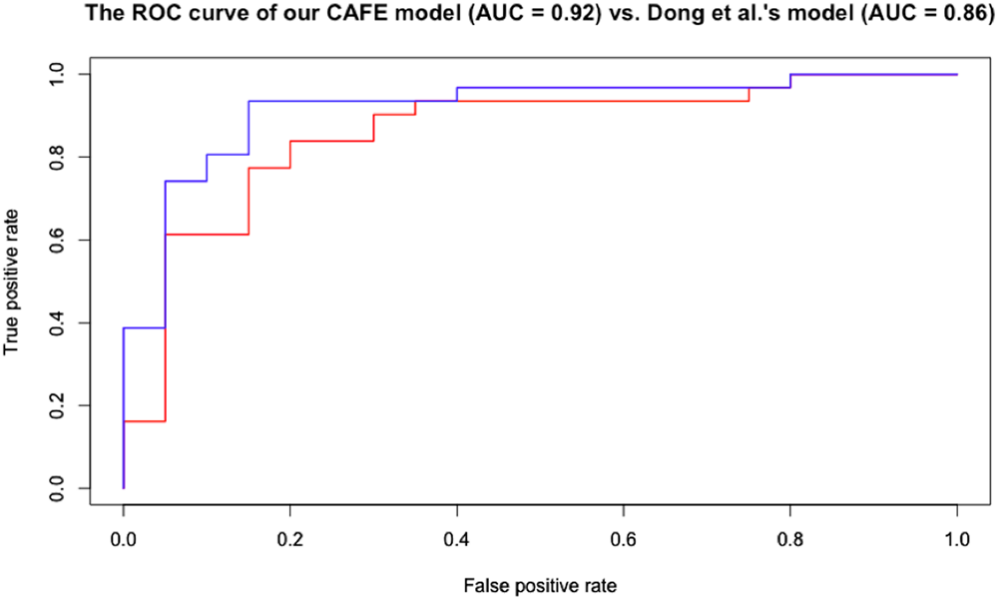
The receiver operating characteristic (ROC) curve of our CAFE model of the combined and optimized L1-regularized and early stopping logistic regression algorithms is graphed in blue. This model achieved a V-score AUC of 0.918. The ROC curve of Dong *et al*.’s model of the L1-regularized logistic regression algorithm is in red. Their model achieved a V-score AUC of 0.858.

As Table 2 shows, CAFE achieved a strong C-score AUC of 0.921, very similar to the C-scores achieved by either individual algorithm. However, it obtained a much higher V-score, which is a substantial improvement when compared with the V-score of 0.858 obtained by Dong *et al*.’s model that did not use active feature extraction. Figure 2 compares the ROC curve of our CAFE model with that of Dong *et al*.

The strong performance from this top-performing CAFE model confirms that it is more robust when facing data variation across hospitals. The AUC of 0.918 demonstrates that the model is a reliable classifier for pathologists to use for real-time decision support.

## Discussion

We first investigate the overall reduced accuracy of the V-score AUC-ROC values in comparison to the C-scores. We applied the CNN model directly to the raw tissue images, so we analyze the implications of using the deep learning algorithm. Finally, we review the findings of our study, particularly in comparison to prior art.

### Analyses of V-scores of DCIS/UDH Classification Models

The V-score of the individual classification models as well as the combined model had a generally reduced accuracy in comparison with the C-score due to variation in image collection mechanisms from different hospitals. Variation across the institution datasets is inevitable due to the different processes for obtaining the images, such as staining, fixating, and scanning the samples. Thus, the V-scores were lower than the C-scores, especially for the random forest and convolutional neural network models (AUC = 0.666 and 0.650, respectively). These specific individual algorithms had mediocre robustness and likely overfit to the training data due to limited dataset size.

### Analyses of V-scores of DCIS/UDH Classification Models with Switched Datasets

To provide a more comprehensive evaluation of the generalizability of the classification models, we switched the training and testing datasets for the V-scores (Table 3). We see similar trends in the performance of Dong *et al*.’s model as well as the L1-regularized LR, random forest, and convolutional neural network models with active features after switching the datasets for training and testing, confirming that the active feature optimization improves diagnosis accuracy. We also noticed an overall reduced accuracy of the models compared to results from the un-switched datasets due to the small training set and the larger testing set. We were unable to determine the V-score with switched datasets for LR with early stopping. The model splits the cases that are not in the testing set into a training set and a validation set and fails to run when the testing set is larger than the training set.

**Table 3:** The performance of Dong *et al*.’s model as well as the L1-regularized LR, random forest, and convolutional neural network models with active features after switching the datasets for training and testing.

Algorithm	V-score for Switched Datasets
L1-regularized LR from Dong *et al*.	0.757
L1-regularized LR with active features	0.827
Random forest with active features	0.662
Convolutional neural network with active features	0.631

### Analyses of Active Features from Validation of DCIS/UDH Classification Models

We analyzed the 28 active features obtained from employing the L1-regularized LR model for validation across hospitals.

### Application of CNN Model to Images

In addition to implementing the six methods to the features dataset, we applied the convolutional neural network model directly to the images to discriminate between DCIS and UDH classes. This algorithm has previously had impressive results on image-based machine learning benchmarks such as MNIST, an image database of handwritten digits^14, 15^. However, even after optimizations and data augmentation, the CNN consistently predicted DCIS for all of the cases. The inability of the network to distinguish between the two classes was likely due to the extremely high variance of the image samples. The number, location, size, and other features of the tumor cells varied widely across samples of the same class, making identification of patterns and significant features difficult. Furthermore, deep learning algorithms tend to perform better on larger datasets and often overfit on smaller datasets.

### Implications of Our Findings

We developed an accurate model to distinguish between DCIS and UDH lesions. By using Dong *et al*.’s features extraction process, our dataset remained unbiased and free of manual intervention. We identified the active features using the L1-regularized logistic regression model, which was made more accurate with the optimal λ. This study is the first to combine models using the various algorithm predictions to obtain a more accurate result for lesion type discrimination. By combining predictions, we developed a more reliable model. For the C-scores of all algorithms, we reduced variance resulting from the fold selection by running the methods 1000 times. Finally, we demonstrated the ability to apply our method to new data from a different hospital, revealing the real-time application across institutions.

Our model can be implemented across multiple laboratories for clinical practice. The algorithm can be used as a second-reader to identify suspicious cases when the pathologist’s diagnosis disagrees with the computational evaluation. Additionally, our methodology of feature extraction and combination of multiple algorithms provides a basis for conducting additional computational research for automated cancer diagnosis. This study can be replicated for further analysis using additional datasets from other hospitals. The optimization methods can be extended to biopsy images, for instance, of the lung, colorectal, and pancreas as well as applied to images for other classification and pattern recognition problems.

### Conclusion and Future Work

Our CAFE model was developed to distinguish the benign (UDH) from the malignant (DCIS) lesions. We optimized our results by reducing test result variance, optimizing the λ parameter, selecting active features, and combining algorithms. These strategies were used for our top-performing model, which achieved a V-score AUC of 0.918, significantly higher at a statistical p-value of 0.01 in comparison with Dong *et al*.’s V-score of 0.858, as well as with results from other studies^6, 16–18^. This increase is noteworthy considering that for every 100 patients, our model would on average correctly diagnose six more cases, preventing these individuals from receiving a potentially harmful mistreatment. Our CAFE model can help pathologists confirm their diagnoses and identify cases that may require additional analysis.

There are a few areas for development of our classification model. Further classification refinement can be made through the extension of our statistical model to discriminate low grade from high grade DCIS. The methods for optimization can also be applied to categorization in other fields of pathology such as classification of muscle weakness grades from ultrasound images^19^ and of the severity of cardiovascular disease from nuclear medicine images^20^ since they also rely on image segmentation and feature extraction.

## Materials and Methods

In this study, we extracted features from patient breast biopsy images from two hospitals. We then input the features dataset into six predictive models of three machine learning classifier types. Finally, we evaluated the algorithms using the area under the curve (AUC) of the receiver operating characteristic (ROC) curve as a score and implemented several optimization methods to improve prediction accuracy.

### Patient Samples and Image Processing

From two different hospitals, we obtained scanned images of breast biopsies of 167 patients, all of whom provided informed consent for study participation. The image dataset includes 80 cases of DCIS and 36 cases of UDH from the Massachusetts General Hospital (MGH), for which the Partners Human Research Committee approved the study. The dataset also includes 20 cases of DCIS and 31 cases of UDH from the Beth Israel Deaconess Medical Center (BIDMC), for which the Beth Israel Deaconess Medical Center IRB approved the study. All methods were performed in accordance with the relevant guidelines and regulations. At both hospitals, the biopsies were processed using standardized procedures: formalin fixed and paraffin embedded tissue was cut into 5 µm sections and stained with hematoxylin and eosin. Per case, one slide per case was digitized using Philips Ultra Fast Scanner 1.6, and one to four diagnostic ROIs were manually selected for image analysis^6^.

We obtained a features dataset using Dong *et al*.’s algorithm on raw tissue sample images, the same as used in this study. After nuclei segmentation was performed using Fiji (ImageJ, National Institutes of Health), the algorithm computed 392 features for each case. Morphological features included geometric and physical measurements such as area, perimeter, and Feret’s diameter, while statistical features included intensity and texture measurements under eight different color channels^6^. All images and data can be found at the following website: http://earlybreast.becklab.org/.

### Machine Learning Algorithms

We used six models from three classifier types to analyze the machine learning algorithm categories that worked best for the classification. The applied algorithms were L1-regularized logistic regression, logistic regression with early stopping, multilayer perceptrons, convolutional neural networks, random forests, and conditional inference forests. These algorithms represent the best-known and mostly widely used machine learning algorithms in the literature^6, 21–30^. Multilayer perceptrons and convolutional neural networks are the two deep learning algorithms, while the other four are categorized as either regression or decision tree-based learning. All the models produced final predictions through probability values on a scale from 0 to 1, with 0 strongly indicating that the sample is UDH, 1 strongly indicating that the sample is DCIS, and 0.5 indicating that the model is uncertain of the class to which the sample belongs. Table 4 summarizes the machine learning models we applied.

**Table 4:** A summary of the various machine learning models that were applied to automate the lesion classification.

Model	Type	Implementation	Layers
L1-regularized LR	Regression	R (glmnet)	2 (input, output)
LR with early stopping	Regression	Python (Theano)	2 (input, output)
MLP	Deep learning	R (neuralnet)	3 (input, hidden layer, output)
CNN	Deep learning	Python (Theano)	5 (filtering, pooling, MLP)
Random forests	Tree-based learning	R (randomForest)	Not applicable
Conditional inference forests	Tree-based learning	R (party)	Not applicable

#### Two Logistic Regression Models

The L1-regularized logistic regression model was implemented in the statistical computing language R with the *glmnet* package in R^31^, while the logistic regression model with early stopping was implemented using the Theano library in Python (https://github.com/Theano/Theano). Both algorithms fit the samples to a logistic curve by minimizing a loss function based on the feature values. The L1-regularized approach minimizes the absolute difference of each feature from its predicted value on the curve, reducing overfitting^32^. On the other hand, the regression algorithm with early stopping splits the samples not used for testing into a training set and a validation set. The model trains on the former set and prevents overfitting through verification on the latter set. Training is ceased when the model no longer improves its score on the validation set.

#### Multilayer Perceptron

The multilayer perceptron (MLP) was implemented with the *neuralnet* package in R^31^. The network consists of a three-layer perceptron containing input, output, and hidden layers. The input layer has 392 nodes, one for each of the features. Our top-performing MLP model contains 30 nodes in the hidden layer, which is the typical number of active features observed in the logistic regression models. The input nodes are connected to the hidden layer nodes with edges, and weights are assigned to these edges to minimize the negative log-likelihood error of the training data. Ideally, the MLP would identify the most significant of the 392 input features and incorporate them in the hidden layer^33^. The nodes in the hidden layer are also connected with weighted edges to two output nodes, corresponding to the likelihood of either UDH or DCIS. The model makes the final prediction between 0 and 1 by dividing the DCIS score by the sum of the UDH and DCIS scores.

#### Convolutional Neural Network

The Convolutional Neural Network (CNN) was implemented in Python using the Theano library^34^. The CNN consists of two convolutional pooling layers that are not completely interconnected as in the MLP. Instead, these layers undergo successive filtering and pooling, which isolate the most significant features in each pooling region and also reduce the variation under translation of the input data^35^. The output from the pooling layers passes through a hidden layer and then an output layer, from which the final predictions are made.

#### Random Forests

**Figure 3:**
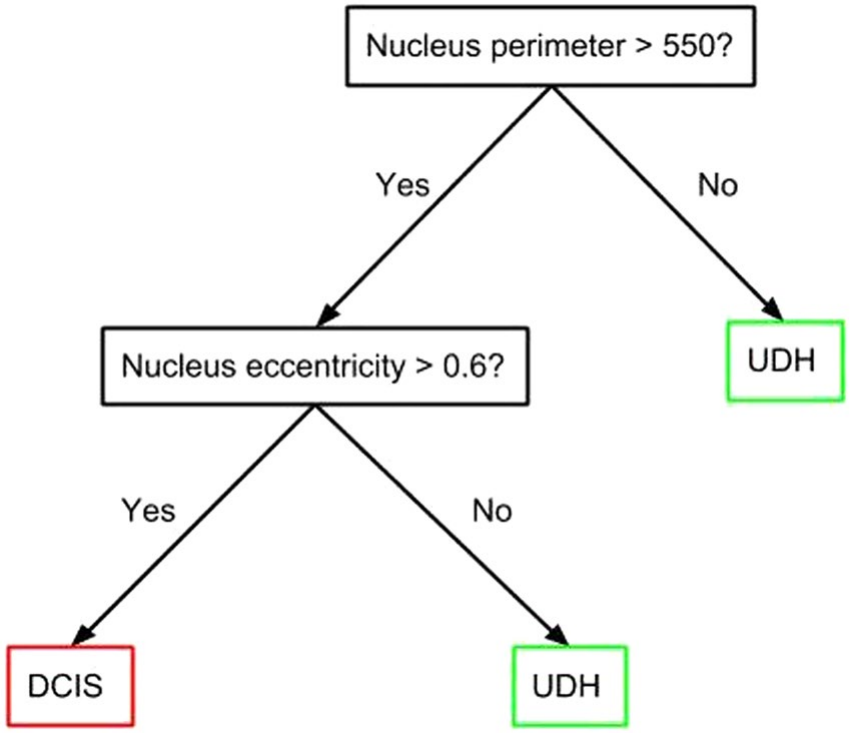
An example decision tree with two features.

The random forest classification system was implemented with the *randomForest* package in R^31^. A random forest is an ensemble of decision trees, each of which is given a subset of *n* total features; see Fig. 3 for an illustration. Although decision trees by themselves are prone to either high variance or bias, many errors counterbalance when compiled into an ensemble^36^. Since each tree is only given a random subset of size $\sqrt{n}$ of the features, all trees are unlikely to become biased in the same manner^36^. The random forest algorithm used in this study computes its predictions by calculating the proportion of 10,000 decision trees that predict either of the two lesion classes for a given sample.

#### Conditional Inference Forests

The conditional inference forest classification system was implemented in R with the *party* package^31^. This model does not consist of standard decision trees but rather of conditional inference trees, which typically use information measures such as the Gini coefficient to determine where to split the tree. Conditional inference trees also utilize multiple significance tests on the permutations of the features on the tree nodes. Ideally, this process helps to reduce some of the bias that can occur in standard decision trees^37^.

### Optimization and Algorithm Development

We implemented various methods to improve prediction accuracy. First, we reduced prediction randomness by establishing a reliable way to measure the accuracies of our models. We noticed that when measuring prediction scores, the randomness caused by seed selection could vary the results by up to 10%. To obtain reliable C-score predictions, we ran each model 1000 times on different splits of the dataset by setting the seed of the random number generator to a different value before each test. We then used the median, which is less prone to outliers and skewness, of the 1000 predictions as the final prediction.

Second, we applied active feature identification and extraction techniques. With a large set of features and a small sample set, we expected dependencies among the features and overfitting when using all the features to train the models. Therefore, we used the L1-regularized logistic regression algorithm to identify active features. We ran this model on all 392 features to obtain the optimal λ value. The features with a weight greater than zero corresponding to this λ value were identified as “active features”. For the V-score predictions, we extracted 28 active features from all features. For the C-score predictions, we ran the logistic regression algorithm 1000 times with different seeds, extracting between 20–40 active features each time. We then ran the six algorithms on the selected feature sets for training and testing and used the median of the 1000 predictions to compute our final scores.

Finally, we combined the predictions of multiple algorithms to improve their accuracy. We observed that if an algorithm gave a correct DCIS/UDH prediction, a similarly performing algorithm tended to give the same prediction, and if it missed, its score was on the borderline. Therefore, we expected the algorithms to reinforce each other when combined. We took the average of continuous prediction scores from multiple algorithms to compute the AUC-ROC value for the combined model. We based our methodology on bootstrap aggregating rather than decision fusion since we used regression models instead of binary classifiers. Thus, we capture more information from the models by averaging the predictions^17^.

Regarding code availability, the Python and R scripts for reproducing validation of our top-performing CAFE model can be accessed using our GitHub repository: https://github.com/evaniradiya-dixit/CAFE-BreastLesionDiagnosis.
